# Polycistronic Expression of the Influenza A Virus RNA-Dependent RNA Polymerase by Using the *Thosea asigna* Virus 2A-Like Self-Processing Sequence

**DOI:** 10.3389/fmicb.2016.00288

**Published:** 2016-03-08

**Authors:** Fumitaka Momose, Yuko Morikawa

**Affiliations:** Laboratory of Viral Infection II, Kitasato Institute for Life Sciences, Kitasato UniversityTokyo, Japan

**Keywords:** self-processing sequence, polycistronic expression, RNA-dependent RNA polymerase, ribonucleoprotein complex, proteasomal degradation

## Abstract

The RNA-dependent RNA polymerase (RdRp) of influenza A virus consists of three subunits, PB2, PB1, and PA, and catalyses both viral RNA genome replication and transcription. Cotransfection of four monocistronic expression vectors for these subunits and nucleoprotein with an expression vector for viral RNA reconstitutes functional viral ribonucleoprotein complex (vRNP). However, the specific activity of reconstituted RdRp is usually very low since the expression level and the ratio of the three subunits by transfection are uncontrollable at single-cell levels. For efficient reconstitution of RdRp and vRNP, their levels need to be at least comparable. We constructed polycistronic expression vectors in which the coding sequences of the three subunits were joined with the 2A-like self-processing sequence of *Thosea asigna* virus (TaV2A) in various orders. The level of PB1 protein, even when it was placed at the most downstream, was comparable with that expressed from the monocistronic PB1 vector. In contrast, the levels of PB2 and PA were very low, the latter of which was most likely due to proteasomal degradation caused by the TaV2A-derived sequences attached to the amino- and/or carboxyl-terminal ends in this expression system. Interestingly, two of the constructs, in which the PB1 coding sequence was placed at the most upstream, showed much higher reporter activity in a luciferase-based mini-genome assay than that observed by cotransfection of the monocistronic vectors. When the coding sequence of selective antibiotic marker was further placed at the most downstream of the PB1-PA-PB2 open reading frame, stable cells expressing RdRp were easily established, indicating that acquisition of antibiotic resistance assured the expression of upstream RdRp. The addition of an affinity tag to the carboxyl-terminal end of PB2 allowed us to isolate reconstituted vRNP. Taken together, the polycistronic expression system for influenza virus RdRp may be available for functional and structural studies on vRNP.

## Introduction

The genome of influenza A virus consists of eight-segmented negative sense, single-strand RNAs (vRNAs) (Portela and Digard, 2002). Both termini of each vRNA form a partially double-stranded structure so-called “panhandle” region, which functions as the transcription promoter and the genome replication origin (Desselberger et al., 1980; Hsu et al., 1987). Each vRNA segment exists as a ribonucleoprotein complex (vRNP) in which viral RNA-dependent RNA polymerase (RdRp) binds the panhandle region, and nucleoproteins (NP) regularly bind the single strand region. This vRNP structure is required for full-length RNA synthesis (Beaton and Krug, 1986; Shapiro and Krug, 1988), the localization of progeny vRNP on Rab11-positive recycling endosome (Amorim et al., 2011; Eisfeld et al., 2011; Momose et al., 2011), high-order assembly of vRNPs, leading to selective packaging of eight progeny vRNP segments into a virion (Hutchinson et al., 2010).

Influenza A virus RdRp is a heterotrimeric complex of PB2, PB1, and PA subunits and is a multifunctional enzyme (Fodor, 2013) that catalyzes both primer-dependent viral mRNA transcription (Plotch et al., 1981; Robertson et al., 1981; Poon et al., 1999; Guilligay et al., 2008; Dias et al., 2009) and primer-independent genome RNA replication (Young and Content, 1971; Hay et al., 1982), the latter of which consists of two distinct steps of RNA syntheses. The first step is the complementary RNA (cRNA) synthesis and the second step is the synthesis of vRNA from the cRNA template for the amplification of progeny vRNP. Structural and functional studies indicate that the amino-terminal (N-terminal) end of PB1, the active center subunit for RNA synthesis, is inserted into the cavity of carboxyl-terminal (C-terminal) domain of PA (He et al., 2008; Obayashi et al., 2008), while the C-terminal domain of PB1 interacts with the N-terminal domain of PB2 (Sugiyama et al., 2009). It has been thought that intermolecular interactions of these subunits and their interactions with host factors are involved in switching the RdRp between transcriptase and replicase and in controlling the 2-step replication of vRNA.

*In vitro* viral RNA synthesis systems with purified RdRp/vRNP (Kawakami and Ishihama, 1983; Honda et al., 1987) have been used to study the function of the viral RdRp, including the promoter structure (Parvin et al., 1989), cap-snatching (Plotch et al., 1979), and host factors required for efficient RdRp activity (Nagata et al., 1989). *In vivo* (cell-based) systems so-called mini-gonome assays, in which a reporter gene is expressed from a model viral genome by RdRp expressed in trans from plasmid vectors or helper virus infection, can be also used to find new RdRp-specific antivirals (Lutz et al., 2005; Ozawa et al., 2013; Wang et al., 2015). For these purposes, stable cell lines expressing viral RdRp have been attempted to be established (Li et al., 1989; Kimura et al., 1992). However, current systems still have limitations and drawbacks and make biochemical analyses difficult to achieve.

One of the limitations is the instability of purified RdRp. The vRNP purified from virions is relatively stable, but further purification using micrococcal nuclease or cesium chloride (Seong et al., 1992), by which NP and vRNA molecules are dissociated, lowers the specific activity of the RdRp (activity per mass). Sole expression of each RdRp subunit in *Escherichia coli* cells is known to result in protein aggregation and *in vitro* reconstitution of the active RdRp complex has not been established. In transfection-based systems, although the expression levels and the ratio of RdRp subunits in total cells can be controlled by the DNA amounts used for transfection, they varied in each cell. If one of the RdRp subunits does not express well in a cell, the other subunits become dead-end products. Indeed, previous reports indicated that serial affinity purifications after reconstitution of vRNP were required to eliminate such dead-end products (Mayer et al., 2007; Jorba et al., 2008). Multi-subunit complexes such as influenza virus RdRp are needed to be correctly assembled for their activity, and this is possible only when all the subunits are equally expressed in individual cells, such as stable cell lines expressing the three subunits. However, it is time-consuming and complicated to select cell clones expressing the three RdRp subunits by the RdRp activity-based screening.

We aimed to efficiently reconstitute multi-subunit complexes such as viral RdRp and vRNP complexes in culture cells. To this end, we constructed a polycistronic expression vector in which three coding sequences (CDSs) of RdRp subunits are concatenated in-frame with the CDS of *Thosea asigna* virus 2A-like self-processing sequence of the capsid precursor protein (referred to as TaV2A) (Pringle et al., 1999; Donnelly et al., 2001a), and reconstituted the active RdRp at single cell levels. The 2A/2A-like peptides are approximately 20 amino acids in length and are encoded in various virus genomes such as picornaviruses (Luke et al., 2008). For this purpose, the 2A sequence of foot-and-mouth disease virus has been studied in most detail (Ryan et al., 1991). The molecular mechanisms of self-processing have been suggested as follows. The translation of self-processing 2A sequence is terminated at the penultimate glycyl residue and restarts at the last prolyl residue without connecting these glycyl-prolyl residues by a peptide bond (Donnelly et al., 2001b). Resultant upstream protein has 2A sequence until the penultimate glycine at the C-terminal end and downstream protein has an additional proline at the N-terminal end after self-processing. In this report, we employ TaV2A-dependent polycistronic expression systems and show efficient reconstitution of functional (biologically active) influenza A virus RdRp. We also discuss not only these advantages, but also the drawbacks about self-processing 2A sequence-based polycistronic expression systems.

## Materials and Methods

### Virus Strain, Antibodies, and Cell Lines

Influenza virus A/Puerto Rico/8/34 (PR8) strain was used throughout this work. Details of rabbit anti-PB2, PB1, and PA antisera (Kawaguchi et al., 2005; Naito et al., 2007b), and anti-NP polyclonal antibody raised against the full length of PR8 NP (Momose et al., 2007) are described elsewhere. A polyclonal antibody specific for the processed form of TaV2A peptide (anti-TaV2Apep, Scrum, Japan) was isolated from rabbits immunized with a corresponding synthetic peptide, cGSLLTaGDVEENPG, with modification of the N-terminal cysteine and internal alanine (lower case c and a, respectively). Human embryonic kidney (HEK293T) and Madin-Darby canine kidney (MDCK) cells were maintained in Dulbecco’s modified Eagle medium (Cat. D5796, Sigma–Aldrich, USA) supplemented 10% fetal bovine serum at 37°C under 5% CO_2_ conditions.

### Construction of v/cRNA and Recombinant Protein Expression Vectors

Construction of the PR8 vRNA expression vectors (pPolI-PR8-x, x = segment number) were essentially described elsewhere (Neumann et al., 1999). At hybridization sites of primers for semiquantitative real-time PCR (qPCR) as shown in our previous report (Momose et al., 2011), PCR-based synonymous substitutions of pPolI-PR8-x were carried out using mutated primer sets (**Supplementary Table S1**). Consequently, neither the modified vectors (pPolI-modPR8-x) nor its transcripts are detectable by using the original qPCR primers. For the standard DNA of the modified segment-specific qPCR, the plasmid (pBSmodPR8qPCRSTD) which contains one copy each of eight amplicons was constructed as previously reported (Momose et al., 2011). For cRNA expression, the cDNA for each cRNA was made using conversion primers (**Supplementary Table S2**, P37–P39) and was cloned into pHH21 (Neumann et al., 1999) (referred to as pPolI-modPR8-Cx) by the In-Fusion cloning system (Clontech Laboratories, USA). As a reporter cRNA expression vector, we used the reverse construct of phPolI-vNS-Luc (Murano et al., 2014) (referred to as phPolI-cNS-Luc) in which the NS1/2 CDSs of the eighth/NS segment cRNA expression vector was replaced with the firefly luciferase CDS.

The CDSs of PB2, PB1, PA, and NP were amplified from pPolI-modPR8-1, -2, -3, and -5, respectively, using specific primer sets (**Supplementary Table S2**). The hygromycin B phosphotransferase (HygR) and *Aequorea coerulescens* GFP (AcGFP) CDSs were derived from pEBMulti-Hyg vector (Wako Pure Chemicals, Japan) and pAcGFP1-Tubulin vector (Clontech Laboratories), respectively. These CDSs were subcloned into phCMV1 (Genlantis, USA), pCAGGS (Niwa et al., 1991), and their derivatives.

**FIGURE 1:**
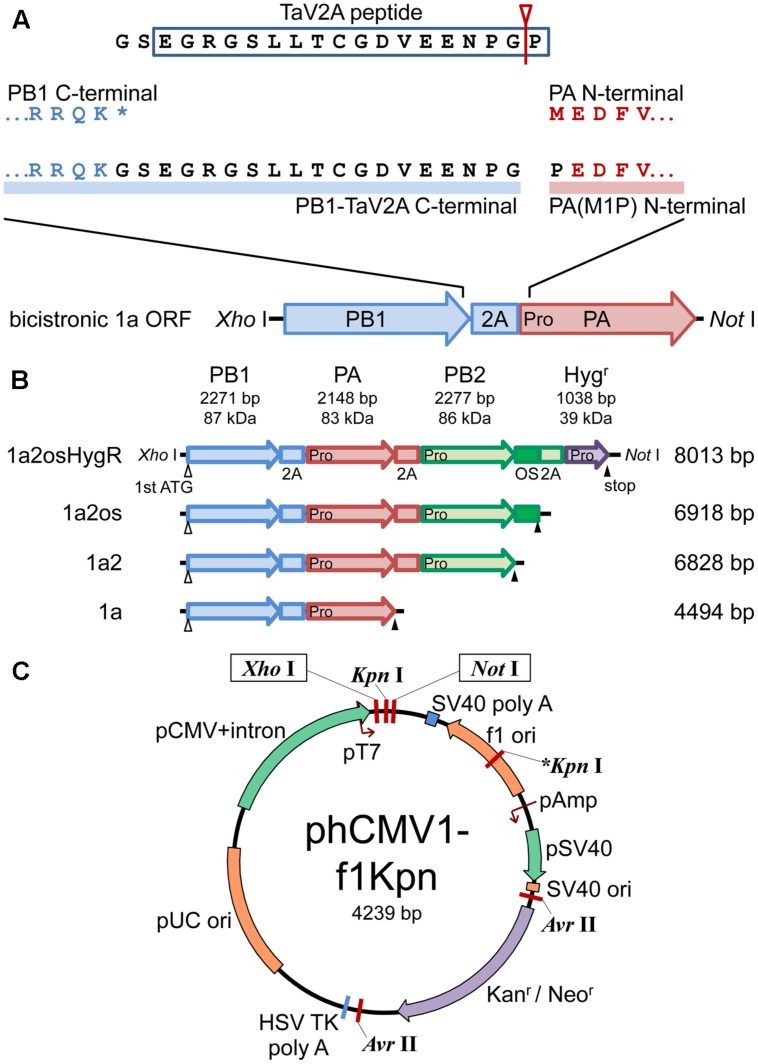
**Schematic representations of polycistronic RdRp expression vectors.** **(A)** Concatenation of CDSs of RdRp subunits with TaV2A self-processing sequence. The bicistronic PB1-PA ORF is shown as an example. The stop codon (^∗^) of the upstream CDS was removed and GS linker-TaV2A peptide CDS was added to the end. Since TaV2A peptide was separated at the Gly-Pro present at the end of the peptide (an arrowhead with a line), the downstream subunit has an additional proline residue at the N-terminal end. Consequently, the original first ATG/methionine codon of the downstream CDS was replaced by proline (M1P substitution, indicated as “Pro” in the PA CDS). **(B)** CDS order of bi-, tri-, and tetra-cistronic ORFs. PB2, PB1, PA, and the hygromycin B phosphotransferase CDSs (abbreviated as 2, 1, a, and HygR, respectively) were concatenated in-frame with TaV2A peptide (2A) and/or One-STrEP/Twin-strep affinity tag (OS) CDSs (Schmidt et al., 2013). The start and end of each ORF are indicated by open (first ATG) and closed (stop) triangles, respectively. TaV2A peptide-derived proline codons/residues are indicated with “Pro” at the beginning of each downstream CDS. The names (left side) and lengths of ORFs (right side) are indicated. **(C)** Plasmid map of phCMV1-f1Kpn, a derivative of phCMV1. Additional *Kpn* I site (indicated as ^∗^*Kpn* I) was created in the f1 *ori* region. The ORFs exemplified in **(B)** were inserted between unique Xho I and Not I sites of phCMV1 or phCMV1-f1Kpn and were transcribed to mRNA under the control of CMV promoter (pCMV). The original phCMV1 vector was routinely used for protein expression. The phCMV1-f1Kpn vector was for establishing stable cell lines expressing RdRp.

For polycistronic expression, concatenation of CDSs was carried out (**Supplementary Figure S1**). Briefly, the CDS of each RdRp subunit was amplified as three types of cDNA for the most upstream, internal, and most downstream positions of an open reading frame (ORF) (**Supplementary Figure S1A**, designated as α, β, and γ fragments, respectively). The ends of each fragment were modified using specific oligonucleotides (**Supplementary Table S2**, P1–P15). A unique *Xho* I site followed by a Kozak sequence (Kz) was added to the upstream end of the α fragment. The authentic stop codons at the downstream ends of α and β fragments were replaced by the TaV2A peptide CDSs (**Supplementary Figure S1D**, TaV2A_1_, TaV2A_2_, and TaV2A_3_ for PB1, PB2, and PA fragments, respectively) with X-TaV2A_n_-GRGSct-rev and TaV2A_n_-g/aGRGS-rev primers. The original TaV2A nucleotide sequences were derived from pDON-5 OKSLN (Takara Bio, Japan) and the TaV2A_1_, TaV2A_2_, and TaV2A_3_ nucleotide sequences contained synonymous substitutions (**Supplementary Figure S1D**) to prevent homologous recombination in host cells. The β and γ fragments lacked the start codon (ATG) using an X-wo1Met-for primer (**Figure 1A**). The γ fragments contained a unique *Not* I site at the downstream end derived from the X-STP-NotI-rev primer. For bicistronic expression, the α-fragments of the PB2, PB1, and PA CDSs were ligated with the γ-fragments for the three CDSs, producing the α–γ fragments in six combinations (**Supplementary Figure S1B**). The α–γ fragments were amplified with a pair of the most upstream and downstream primers, XhoI-Kz-X-for and X-STP-NotI-rev primers, and were cloned into phCMV1 (referred to as phCMV-mod2P-αγ). For tricistronic expression, the α–β and β–γ fragments were made (**Supplementary Figure S1B**) and overlap PCR was carried out by using the fragments as templates and a pair of most upstream and downstream primers (**Supplementary Figure S1C**). The resultant αβγ PCR products were similarly cloned into phCMV1 (referred to as phCMV-mod3P-αβγ).

For affinity purification, the Twin-Strep tag (formerly known as the One-STrEP tag, IBA, Germany) sequence (Schmidt et al., 2013) was inserted at the downstream end of the PB2 CDS in-frame with relevant primers (**Supplementary Table S2**, P16–P18), and the product was referred to as phCMV-mod3P-1a2os. For stable cells expressing the RdRp subunits, the HygR CDS was placed at the most downstream of the monocistronic and polycistronic ORFs in phCMV1-f1Kpn, a phCMV1 derivative containing an additional *Kpn* I site in the f1 *ori* (**Figure 1C**) by the In-Fusion cloning system using relevant primers (**Supplementary Table S2**, P19–P36). The resultant plasmids were referred to as phCMV-X-HygR.

*Escherichia coli* Mach1 or DH10B strain (Life Technologies, USA) was routinely used for transformation with expression vectors. In the case of RdRp subunit expression vectors, an *E. coli* Mach1 or HB101 strain was used and cultured at 30°C for 24–36 h because of temperature-sensitive growth, as described in the Section Discussion.

### DNA Transfection and Western Blotting Analysis

Equimolar plasmid vectors, pCAGGS (for NP expression), phCMV (for RdRp subunits expression), and pPolI (for cRNA expression), were mixed and were transfected to HEK293T or MDCK cells. Briefly, total N μg DNA and 2.5 × N μl of Lipofectamine 2000 (Life Technologies) were separately diluted in 25 × N μl of Opti-MEM I (Life Technologies) and were incubated for 5 min. These solutions were mixed and incubated for 30 min. The mixtures were inoculated to 5 × 10^5^ cells. In some experiments, MG132 (Calbiochem, USA) was added at 20 μM. At 24 h post-transfection (hpt), cells were harvested for Western blotting analysis and mini-genome assay. For stable cell lines, the medium was replaced with selective medium including the neomycin analog (G418) or hygromycin B (HygB) (Nacalai Tesque, Japan).

For Western blotting, protein samples were separated by SDS-PAGE (7.5% gel), transferred to PVDF membrane, and probed with rabbit anti-PB2, -PB1, -PA antisera, and anti-NP polyclonal antibody. Chemiluminescence detection was carried out by using the ECL or ECL Prime reagent (GE healthcare, UK). Polycistronically expressed proteins harboring processed-TaV2A peptide were detected with rabbit anti-TaV2Apep polyclonal antibody.

### Mini-Genome Assay

HEK293T cells were cotransfected with the reporter cRNA expression vector and various combinations of RdRp and NP expression vectors. At 24 hpt, cells were harvested and luciferase activity was measured in relative light units (RLUs) by using ONE-Glo Luciferase Assay System (Promega, USA). The RLU of each sample was indicated as folds relative to the RLU of control sample using three monocistronic expression vectors for RdRp.

### Establishment of Stable Cells and Cell Lines Expressing RdRp

Following transfection, MDCK cells were cultured for 2 weeks with occasional passages in the presence of 800 μg/ml of G418 or 400 μg/ml of HygB. For stable cell lines expressing RdRp, cell cloning was carried out by limiting dilution in the presence of HygB. TaV2A peptide-positive clones were isolated after indirect immunofluorescence microscopy with anti-TaV2Apep antibody. In each cell clone, fluorescent images for RdRp were acquired by conventional fluorescence microscope (BZ-8000, KEYENCE, Japan) and the expression levels of approximately 50 cells were semiquantified by using ImageJ software (Schneider et al., 2012). The clone No. 5, with the highest level of RdRp expression, was used for observation of cell shapes and cell cycle analyses by Cell-Clock assay (Biocolor, UK) and flow cytometry.

### Flow Cytometric Analysis

Madin-Darby canine kidney cells were detached with trypsin-EDTA and washed with PBS. Cells were fixed with 4% paraformaldehyde and were immunostained with rabbit anti-TaV2Apep antibody and Alexa Fluor 488-conjugated anti-rabbit Ig secondary antibody (Life Technologies). For cell cycle analysis, cells were fixed with chilled 70% ethanol over 4 h. After rehydration with PBS containing 1% BSA, immunofluorescent staining was similarly carried out. Cells were treated with 100 μg/ml RNase A at 37°C for 30 min and stained with 25 μg/ml propidium iodide (PI, Nacalai Tesque). By flow cytometer (FC 500, Beckman Coulter, USA), forward- and side-scatters with linear amplification (FS- and SS-Lin, respectively), fluorescence intensity of Alexa Fluor 488 with logarithmic amplification (FL1-Log) were collected. For cell cycle analysis, fluorescence intensity of the PI with linear amplification (FL3-Lin) and its peak value (AUX) were also collected. Signal analyses were carried out by using CXP software (Beckman Coulter) or Flowing Software version 2.5.1 (www.flowingsoftware.com). TaV2A peptide-positive events were separated from 1∼2 × 10^4^ total events on a dot plot of FS-Lin versus FL1-Log. For cell cycle analysis, singlet events were chosen from 1 × 10^5^ total events on a dot plot of FL3-Lin versus AUX. G0/G1, S, and G2/M regions on a FL3-Lin histogram were automatically separated by using a region control tool of Flowing Software for cell cycle analysis.

### Reconstitution and Isolation of vRNP

HEK293T cells (5 × 10^6^ cells) were cotransfected with phCMV-mod3P-1a2 or -1a2os (polycistronic 1a2 or 1a2os ORF expression vector), pCAGGS-modPR8-NP, and pPolI-modPR8-C6. At 24 hpt, cells were washed with cold PBS and were lysed with 1 ml of NET buffer (20 mM Tris-HCl (pH 7.9), 0.5 M NaCl, and 1 mM EDTA) containing 0.5% Nonidet P-40 (NETN) supplemented with 1 mM DTT, and protease inhibitor cocktail (Cat. No. 25955-11, Nacalai Tesque). After centrifugation at 17,400×*g* for 5 min, the supernatant (input lysate) was used for affinity pull-down assay. Ten microliter packed volume of MagStrep type 2HC beads (IBA) was incubated with 500 μl of NETN containing 40 or 400 μl of input lysate, 1 mM DTT, 100 ng/μl of BSA, and protease inhibitor cocktail, for 60 min at 4°C by using an orbital rotator and was washed with NETT (NET buffer containing 0.5% Tween-20) for 5 min twice. The beads were divided into aliquots for SDS-PAGE followed by Western blotting and for qPCR for viral RNAs, as reported previously (Momose et al., 2011).

## Results

### Construction of Polycistronic RdRp Expression Vectors

To construct polycistronic ORFs, the first methionine and stop codons of each CDS for RdRp subunits were removed and the three CDSs were joined in-frame with di-peptide linker (GS) followed by a TaV2A peptide (EGRGSLLTCGDVEENPGP) CDSs in six different subunit CDS orders (**Figure 1** and **Supplementary Figure S1**). Resultant subunits expressed from the most upstream and the internal CDSs harbored additional 19 residues of GS linker and processed form of TaV2A peptide at the C-terminal ends, and the first methionine codons of subunits expressed from the internal and the most downstream CDSs were substituted by proline derived from the last residue of TaV2A peptide (M1P substitution) (**Figure 1A**). We used phCMV-1 (Genlantis), a small mammalian expression vector (**Figure 1C**). phCMV1-f1Kpn, a phCMV-1 derivative in which an additional *Kpn* I site was created in the f1 *ori* region, was also used to linearize the vector. The polycistronic ORFs were inserted between the unique *Xho* I and *Not* I sites of the expression vectors (12 kbp maximum). In the same manner, bicistronic, and monocistronic vectors were also created.

### Reconstitution of Functional RdRp and vRNP

**FIGURE 2:**
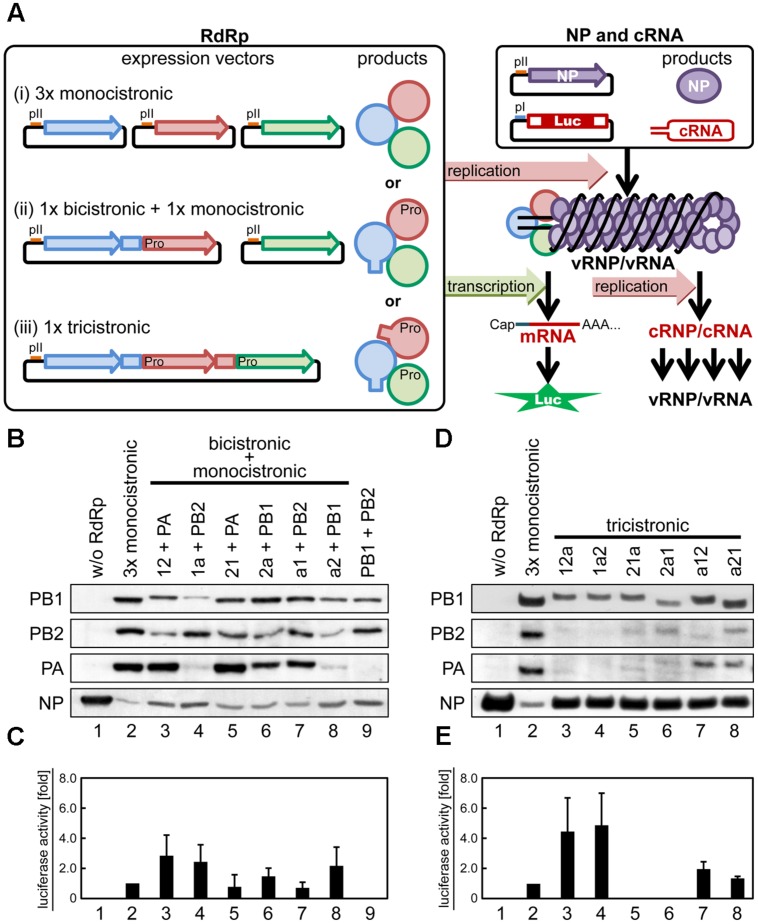
**Reconstitution of functional vRNP by various combinations of the RdRp expression vectors.** **(A)** Schematic representation of structures of expression vectors for a mini-genome assay. In HEK293T cells, three subunits of RdRp are transiently expressed from (i) three individual monocistronic vectors, (ii) a combination of one bicistronic and one monocistronic vectors, or (iii) a tricistronic expression vector. In polycistronic expression, processed TaV2A peptide at the C-terminal ends of the most upstream and internal CDS products are depicted as rectangles protruded from circles, and the proline residues at the N-terminal ends of the internal and most downstream CDS products are indicated as “Pro”. RdRp and NP are expressed under the control of RNA polymerase II promoters (pII, red lines). A positive-strand cRNA is transcribed under the control of an RNA polymerase I promoter (pI, blue line). The reporter cRNA contains the firefly luciferase CDS (Luc) flanked by untranslated regions of influenza A virus NS segment in positive sense. **(B–E)** Representative Western blots **(B,D)** and mini-genome assays **(C,E)** in combinations of bicistronic and monocistronic vectors **(B,C)** and tricistronic RdRp expression vectors **(D,E)**. Lanes in **(B,D)** correspond to columns in **(C,E)**, respectively. Viral proteins were detected with rabbit anti-PB2, -PB1, and -PA antisera and anti-NP antibody **(B,D)**. Reporter luciferase activities were measured in RLU and the average with standard deviation from four independent experiments are shown as folds. The RLU of control sample (3 × monocistronic) was set at 1.0 in each experiment.

To estimate the reconstitution efficiency of functional RdRp, we employed a mini-genome assay. HEK293T cells were cotransfected with various combinations of polycistronic and monocistronic expression vectors for RdRp (**Figure 2A**, left side) plus the NP and the model genome expression vectors (right side). In our mini-genome assay, the model viral RNA is transcribed as a positive-strand cRNA under the control of an RNA polymerase I promoter (**Figure 2A**). Primer-independent negative-strand RNA synthesis (cRNA to vRNA replication) occurs by the reconstituted RdRp, resulting in the formation of the vRNP complex. This reconstituted vRNP is used for the primer-dependent mRNA synthesis catalyzed by the RdRp (vRNA to mRNA transcription), leading to expression of luciferase as a reporter. The reconstituted vRNP is also used for the next step of replication, primer-independent positive-strand RNA synthesis (vRNA to cRNA replication). The use of cRNA as an initial RNA assures that the reconstituted RdRp is functional and has both replication and transcription activities.

The expression of RdRp subunits and NP was detected by Western blotting (**Figures 2B,D**). The reconstitution of functional RdRp/vRNP complex was assessed by luciferase activity (**Figures 2C,E**). The expression levels of RdRp subunits, especially PB2 and PA, from polycistronic expression vectors were low, as compared with those expressed from monocistronic vectors (**Figures 2B,D**, compare lanes two and 3–8). It is likely that the translation resume at the TaV2A sequence of the polycistronic mRNA was inefficient and/or that the protein with the TaV2A-derived residues was unstable.

Mini-genome assays (**Figures 2C,E**) showed that the luciferase activities were detected only in the presence of all three RdRp subunits (columns 2–8) but that the levels varied, depending on the combination of vectors and the subunit CDS orders. In tricistronic ORF expression, PB1 was clearly detected even though the protein levels were slightly lower than that produced by monocistronic PB1 expression, whereas the levels of tricistronically expressed PB2 and PA were very low or below the detection limit (**Figure 2D**). Mini-genome assays, however, revealed that the luciferase activities were not similar levels in the tricistronic ORF vectors (**Figure 2E**). The luciferase expression by the PB1-PA-PB2 and PB1-PB2-PA (referred to as 1a2 and 12a) ORF vectors markedly increased up to four to fivefolds (**Figure 2E**, columns 3 and 4). The luciferase expression by the a12 and a21 ORF vectors (columns 7 and 8) was equivalent to that of the control sample and that by the 21a and 2a1 ORF vectors was not detected (columns 5 and 6). These results indicated that the quantity of functional RdRp complex reconstituted in cells did not correlate with the protein levels of RdRp subunits expressed in the cells.

More importantly, these observations suggest that the reconstitution efficiency of functional RdRp complex is greatly influenced by the most upstream CDS of a tricistronic ORF. This trend was also observed in combinations of bicistronic and monocistronic vectors. A comparison of the 12 and 21 ORF vectors (**Figure 2B**, 12+PA and 21+PA) indicated that PB1 CDS at the upstream resulted in the increase of reporter expression by threefold (**Figure 2C**, compare columns 3 and 5). A similar increase was observed for the 1a and a1 ORF vectors (**Figures 2B,C**, columns 4 and 7). Taken together, these results suggest that the PB1 CDS at the most upstream of polycistronic ORF is preferable for efficient reconstitution of functional RdRp. Both PA and PB2 CDSs were tolerant at the most downstream in tricistronic ORFs (**Figure 2E**, compare columns 3 and 4), suggesting that addition of the 19 residues of the linker and processed TaV2A sequence to the C-terminal end of PB2 or PA does not prevent reconstitution of functional RdRp.

### Proteasomal Degradation of the PA Protein Expressed from the Downstream of TaV2A Sequence

Tricistronic RdRp expression systems allowed for efficient reconstitution of functional RdRp with three subunits. Although the levels of PB2 and PA were sufficient for catalytic assays such as a mini-genome assay, they appeared insufficient for purification of reconstituted vRNP as protein sources such as structural studies. To improve the yield of functional RdRp, we tested the following three methods: One was a mutagenic approach by which the expression of the downstream protein was expected to be improved to the stoichiometric amounts. Another was the establishment of stable cell lines expressing RdRp to ensure the RdRp expression in all cells without transfection. The other was increasing the scale of cell culture followed by affinity purification.

**FIGURE 3:**
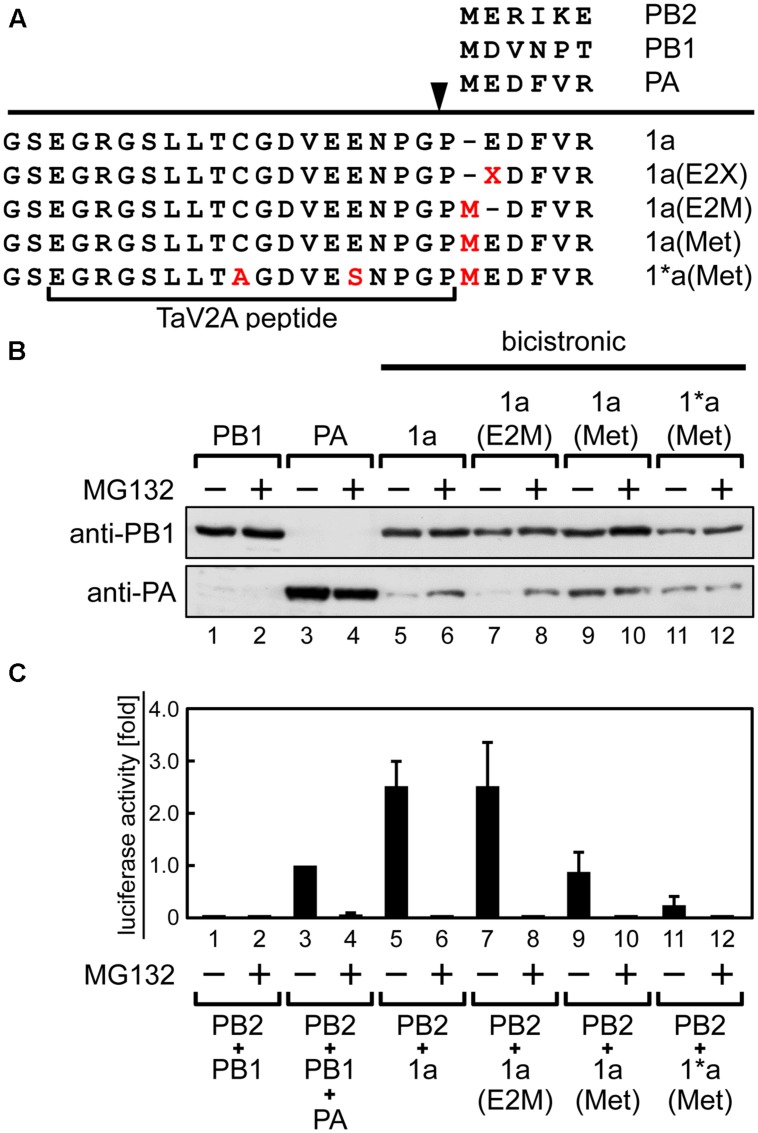
**Stabilization of the downstream PA expressed from the 1a ORF by amino-terminal mutation or treatment with proteasomal inhibitor MG132.** **(A)** Representation of the bicistronic 1a ORF vector with mutations at the TaV2A and PA N-terminal sequences. The processing site of TaV2A peptide is indicated as an arrowhead. Amino acid substitutions C9A and E14S in TaV2A peptide, E2X or E2M in PA, and insertions M in PA are indicated in red. E2X indicates substitution of the second glutamate residue of the downstream PA. N-terminal sequences of each RdRp subunit are shown at the top. **(B)** Western blotting analysis of the downstream PA expression. HEK293T cells were transfected with monocistronic vectors (lanes 1–4), the bicistronic 1a ORF vector (lanes 5 and 6), and its derivatives (lanes 7–12) and were incubated in the absence or presence of 20 μM MG132. PB1 and PA were detected using rabbit anti-PB1 and anti-PA antisera, respectively. **(C)** Mini-genome assays using monocistronic and bicistronic RdRp expression vectors in various combinations. RdRp expression vectors were cotransfected with NP and reporter cRNA expression vectors to HEK293T cells. After 24-h incubation in the absence or presence of MG132, luciferase activities were measured. The fold increase of RLU is shown as average with standard deviation in three independent experiments. The RLU of control sample (lane 3, PB2+PB1+PA) was set at 1.0 in each experiment.

First, using the bicistronic 1a ORF vector (**Figure 1A**), we introduced amino acid deletions or substitutions into the TaV2A sequence and explored whether the inefficient downstream translation was rescued by the mutations (**Supplementary Figure S2**). However, none of the mutants we tested showed any improvement of the downstream PA expression. It has been known that the type of residues (e.g., F, W, Y, L, I, R, K, and H) at the N-terminal end significantly reduce the protein half-life (Varshavsky, 1997). In the 1a ORF construct, the downstream PA expressed from this bicistronic vector contained a M1P substitution (**Figure 1A**). We speculated that this unique mutation decreased PA protein half-life. In fact, the downstream PA was stabilized in the presence of a proteasome inhibitor MG132 (Lee and Goldberg, 1998) (**Figure 3B**, lane 6; **Supplementary Figure S3**, lane 4), indicating that the apparently decreased expression of the downstream PA was likely due to the M1P substitution but not to inefficient translation resume at the TaV2A sequence. Then, we modified the N-terminal end of the downstream PA. Because the first proline cannot be changed in the 2A/2A-like sequence self-processing system, we substituted the second glutamate (E2X) which possibly affected the protein half-life. However, none of E2X mutants we tested showed any improvement of protein stability (**Supplementary Figure S3**). The only case showing stabilization of the downstream PA was that the original first methionine was restored between N-terminal end proline and the second glutamate, i.e., 1a(Met) ORF vector (**Figure 3B**, lane 9), resulting in one proline extension to the N-terminal end of wild-type PA sequence (**Figure 3A**).

Thus, the protein stability was improved by using the 1a(Met) mutation or a proteasome inhibitor. However, the reconstitution efficiency of functional RdRp did not correlate with the stability of the downstream PA. When we reconstituted RdRp using the bicistronic 1a(Met) ORF vector with the monocistronic PB2 vector, the luciferase activity became lower than that by the original 1a ORF and the monocistronic PB2 vectors (**Figure 3C**, compare columns 5 and 9). This result suggests that only one addition of proline at the N-terminal end of PA resulted in a significant decrease of the reconstitution efficiency of functional RdRp. We found that treatment with MG132 abolished the luciferase expression (**Figure 3C**, even number columns), consistent with a previous study in which RNA synthesis of influenza virus was inhibited in the presence of MG132 (Widjaja et al., 2010).

### Establishment of Stable Cell Lines with Polycistronic Expression Vector

Next, we attempted to establish stable cell lines expressing RdRp. Cotransfection of the monocistronic expression vectors for each subunit requires selection of cell clones by three different resistant markers to ensure expression of the three subunits otherwise by RdRp activity-based screening. In contrast, the use of polycistronic expression vector with a single selection marker was expected to allow for expression of all the three subunits in individual cells, leading to the efficient reconstitution of functional RdRp. Furthermore, it is often experienced that when a target gene and a drug resistance gene are coexpressed from different loci, the expression of the target gene is suppressed despite acquisition of the drug resistance. Thus, we added the HygR CDS to the downstream of the polycistronic and monocistronic ORFs (**Figure 1B**). The acquisition of HygB resistance by HygR ensures the expression of the upstream RdRp CDSs.

**FIGURE 4:**
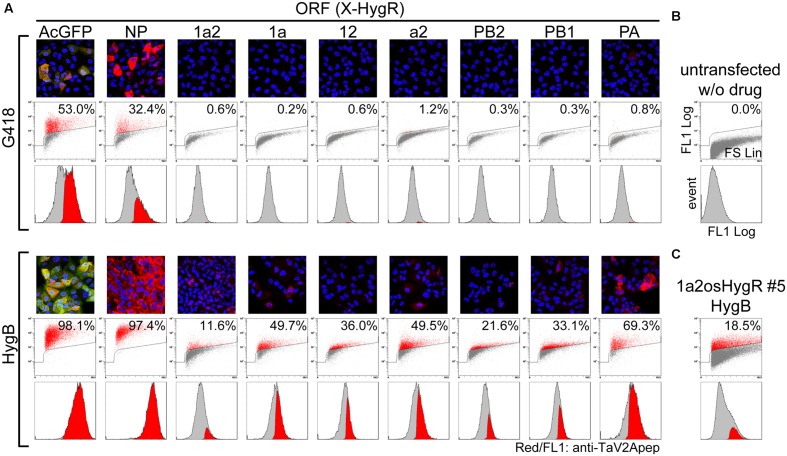
**Improvement of the RdRp-positive cell ratio by polycistronic expression of a selection marker from the most downstream of ORF.** **(A)** Measurement of the RdRp-positive cell ratios. MDCK cells were transfected with unlinearized phCMVK-X-HygR polycistronic expression vectors (X is indicated above a column). Following drug selection in the presence of G418 or HygB (upper and lower panels, respectively) for 2-weeks, indirect immunofluorescence microscopy (top images) and flow cytometry (middle plots and bottom histograms) were carried out using anti-TaV2Apep antibody (FL1, shown in red). In fluorescent images, nuclear DNA and AcGFP were also shown in blue and green, respectively. Axes of flow cytometric plots were indicated in **(B)** and the parameters were described in the Materials and Methods section. Middle dot plots, cell size (FS Lin) versus TaV2A peptide content (FL1 Log) with a polygon gate and the ratio of TaV2A peptide-positive cells (red). Bottom histograms, the TaV2A-peptide content (FL1 Log) versus cell counts (events), in which TaV2A peptide-positive events (red) are superimposed on the total events (gray). **(B)** Flow cytometric analyses of untransfected MDCK cells (negative control). **(C)** MDCK cells were transfected with linearized phCMVK-1a2osHygR and TaV2A peptide-positive clones were isolated by limiting dilution in the presence of HygB (**Supplementary Figure S4**). After several passages, the clone No. 5 was analyzed by flow cytometer.

Madin-Darby canine kidney cells were transfected with the polycistronic vectors. First, we screened in the presence of G418 for the neomycin resistance gene, which was expressed from the locus other than the polycistronic ORFs (**Figure 1C**). When the drug-resistant cell populations were obtained and analyzed by indirect immunofluorescence microscopy using anti-TaV2Apep antibody, the frequency of TaV2A peptide-positive cells was low (**Figure 4A**, G418). Flow cytometry revealed that the percentage of AcGFP-expressing cells was 53.0% and that of cells expressing NP, one of vRNP components, was 32.4% (compare with **Figure 4B**). However, the percentages of cells expressing the RdRp subunit(s) were extremely low (maximum 1.2% in the a2-HygR ORF case).

Then, we carried out cell selection with HygB. As shown in **Figure 4A** (HygB), the percentages of AcGFP- and NP-positive cells markedly increased to 98.1 and 97.4%, respectively. The percentages of cells expressing the RdRp subunits also increased to 11.6–69.3% when compared with the rate by G418 selection. Among these RdRp cases, there was a trend that the positive rate was relatively high when the ORF contained the PA CDS (1aHygR, a2HygR, and PAHygR).

Next, we carried out cloning of the stable cell lines. We constructed 1a2HygR ORF derivatives in which the One-STrEP/Twin-Strep tag CDS (os) (Schmidt et al., 2013) was inserted between the PB2 CDS and the HygR CDS to allow affinity purification (**Figure 1B**). The 1a2osHygR ORF vector was linearized with *Kpn* I at the f1 *ori* region and was transfected to the MDCK cells. The HygB-resistant cell population was selected and was subjected to limiting dilution cloning to establish stable cell lines expressing RdRp (**Supplementary Figure S4**). Despite cell cloning, the expression level of RdRp in each cell varied, ranging from strongly positive [mean fluorescence intensities (MFI) >200] to below the visual detection limit (MFI <100). Clone No. 5 was composed of cells with relatively high level of expression. After several additional passages, we measured again the RdRp expression level by flow cytometer (**Figure 4C**). The positive rate of 1a2osHygR clone No. 5 was slightly higher (18.5%) than that of the heterogenous 1a2HygR cell population (11.6%). It is possible that the polycistronic RdRp-HygR expression tends to be quickly silenced to a minimum expression level required to maintain drug resistance.

Since the growth of HygB-resistant cells expressing all RdRp subunits was extremely slow, we explored the possibility of cell cycle arrest by using Cell-Clock Assay (Biocolor), a colorimetric detection of the cell cycle phase (**Supplementary Figure S5A**) and by measuring DNA content by flow cytometry (**Supplementary Figures S5B,C**). Although results did not show the cell cycle arrest in the RdRp expressing cells, this cell line displayed aberrant cell morphology such as cell enlargement (**Supplementary Figure S5A**, left side), confirming high forward scatter in flow cytometry (**Supplementary Figure S5B**, FS Lin vs FL1 Log of RdRp).

### Isolation of Reconstituted vRNP Complex

We used a large volume of the cell lysates, in which RdRp was polycistronically expressed, and attempted to isolate the reconstituted single segment vRNP. To this end, single polycistronic expression vector (1a2 or 1a2os ORF) or three monocistronic vectors (PB2os, PB1, and PA) for the RdRp subunits were cotransfected with the NP and cRNA expression vectors into HEK293T cells. The cell lysates were subjected to streptavidin-based affinity pull-down assay. The viral proteins (PB2, PB1, PA, and NP) and vRNA were eluted from the affinity beads and were analyzed by Western blotting and polarity-specific qPCR, respectively. In this polycistronic expression system, we used cRNA as an initial RNA transcribed from the RNA expression vector (**Figure 2A**), thereby ensuring the polarity-specific vRNA quantification.

**FIGURE 5:**
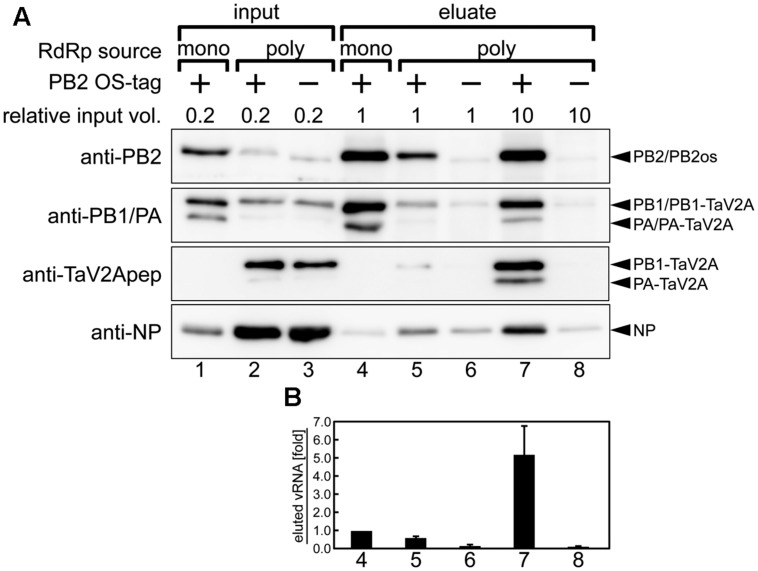
**Affinity pull-down assay of reconstituted vRNP.** **(A)** Western blotting analyses of input lysates (lanes 1–3) and eluates from affinity beads (lanes 4–8) using rabbit anti-PB2, -PB1, -PA antisera, anti-TaV2Apep, and -NP antibodies. vRNP components were expressed and reconstituted by cotransfection of the RdRp expression vectors (poly, phCMV-mod3P-1a2 or -1a2os; mono, phCMV-modPR8-PB2os, -PB1, and -PA) with NP and segment 6 cRNA expression vectors (pCAGGS-modPR8-NP and pPolI-modPR8-C6, respectively) in the HEK293T cells. The relative volume of input lysate used in each lane was shown. The 1a2 ORF vector, not containing affinity tag, was used as a negative control (indicated as minus). Blots are derived from a single pull-down experiment and are representative of three independent experiments. **(B)** Quantitation of coprecipitated vRNA. Each column corresponds to the same lane as shown in **(A)**. Polarity-specific reverse transcription was carried out using the modified segment 6-specific primer for vRNA (**Supplementary Table S1**). Semiquantitative real-time PCR was carried out by using the modified segment 6-specific primer set with standard DNA, pBSmodPR8qPCRSTD. The fold increase of coprecipitated vRNA is shown as average with standard deviation in three independent experiments. The vRNA level of monocistronic expression sample (lane 4) was set at 1.0.

When equal volumes of lysates were used for this affinity pull-down, the level of PB2os extracted from the polycistronic RdRp expression sample was about 10-fold lower than that of the monocistronic expression sample (**Figure 5A**, anti-PB2, compare lanes 4 and 5). Since our polycistronic expression system did not produce high levels of PA and PB2os, we used large volumes of the cell lysates and compared the coprecipitation efficiencies of vRNA and NP per RdRp with the efficiency obtained by monocistronic expression system. When 10-fold excess volumes of the polycistronically expressed cell lysates were used for affinity pull-down, the level of extracted PB2os was comparable to that obtained from the monocistronically expressed cell lysates (**Figure 5A**, anti-PB2, compare lanes 4 and 7). In this condition, the levels of coprecipitated PB1 and PA, were also comparable (anti-PB1/PA, lanes 4 and 7). From these results, the levels of precipitated RdRp were considered as nearly equivalent. A careful observation indicated, however, that only a trace of NP was coprecipitated in the monocistronic expression system (anti-NP, lane 4). The RdRp consists of equimolar PB2, PB1, and PA subunits and the vRNP complex contains one set of RdRp, one copy of viral RNA and excess molar of NP. It is possible that the RdRp complex but not the vRNP complex was preferentially formed in the monocistronic expression system.

Consistent with these findings, the coprecipitated vRNA levels in the polycistronic expression was fivefold higher than that observed in the monocistronic expression when similar levels of RdRp were isolated (**Figure 5B**, compare columns 4 and 7), indicating that vRNA formed vRNP complex more efficiently when RdRp complex was reconstituted by polycistronic expression. From these results, we concluded that, though 10-fold volume of cell lysate was required to obtain an equal quantity of RdRp, the polycistronic RdRp expression system allowed for the reconstitution of vRNP complex and for the isolation of the complex by one-step affinity purification. Thus, our self-processing 2A-based polycistronic expression may be good for biological analysis requiring enzymatically functional RdRp expression, such as a mini-genome assay and a reverse genetic approach, but not suitable for analysis requiring a large quantity of reconstituted vRNP.

## Discussion

Our present study displayed the reconstitution of functional RdRp by the polycistronic expression of three RdRp subunits with TaV2A self-processing sequence. This expression system allowed us to obtain reconstituted vRNP with less purification steps and also to establish stable cell lines expressing RdRp, a viral multi-subunit complex. We suggest that the efficient reconstitution of RdRp and vRNP may allow more detailed functional analyses such as the residues of RdRp necessary for viral transcription and RNA genome replication for full understanding of the influenza virus multiplication. A higher specific activity of vRNP (**Figure 2**) achieved by an efficient reconstitution (**Figure 5**), may allow us to increase the sensitivity of drug screenings such as mini-genome assays. The use of the stable cell lines expressing RdRp will make cell-based antiviral screens possible. It is also possible to screen the host factors necessary for functional RdRp/vRNP formation and viral RNA synthesis. Here we discuss the pros and cons of the polycistronic expression system.

### Advantages of the Polycistronic Expression by Using a 2A/2A-Like Self-processing Sequence

A variety of expression systems for the influenza virus RdRp and vRNP complexes have been reported, and each system has both advantages and disadvantages. If a large quantity of RdRp is required for studies, such as a crystal structure analysis, the expression systems using insect viruses are advantageous (Reich et al., 2014; Hengrung et al., 2015). The use of Drosophila or yeast cells allows us to find host cell factors involved in viral RNA synthesis by virtue of their genetic knowledge (Naito et al., 2007a; Hao et al., 2008). However, it is uncertain whether the RdRp/vRNP complex reconstituted in these cells are functionally identical with that reconstituted in a mammalian cells. In contrast, MDCK and HEK293T cells are infection-permissive and have commonly been used to reconstitute these multi-subunit complexes by cotransfection of monocistronic expression vectors. However, as shown in this study, the reconstitution efficiency of functional RdRp/vRNP complex was considerably low because the ratio of RdRp subunits varies in each cell.

One advantage of self-processing 2A-based polycistronic expression is to ensure coexpression of multiple proteins in a single cell, suggesting potential applications requiring the biological quality, i.e., the uniformity and reproducibility of the expression ratio, rather than the quantity. By this approach, we explored the reconstitution of functional RdRp complex and vRNP complex with a TaV2A-based polycistronic RdRp expression vector. The reconstitution efficiency of RdRp in the polycistronic expression system was higher than that in the monocistronic expression system (**Figure 2**). If the activity of RdRp was estimated only from protein expression levels in **Figure 2D**, it would be expected that the monocistronic expression sample (lane 2) harbors much higher reporter activity than the others. However, we found that it was not the case. Even though PB2 and PA levels were below the detection limit, polycistronically expressed RdRp from the 12a and 1a2 ORF vectors showed much higher activities (**Figure 2E**, lanes 3 and 4). This result implies that most of the monocistronically expressed proteins probably exist as incomplete/non-functional complexes or monomers and that the contamination of such defective complex make it difficult to purify the target complex. The improvement of the RdRp reconstitution efficiency allowed us to isolate reconstituted vRNP from a crude cell lysate with lesser purification steps (**Figure 5**). In our polycistronic expression, the levels of PB2 and PA were still low, as compared with those observed in the monocistronic expression, but the reconstitution of vRNP was considerably efficient. We suggest that if the polycistronic expression of downstream PB2 and PA could be stabilized, the reconstitution efficiency would more increase.

Internal ribosomal entry site (IRES) has been also used for multiple protein expression from one mRNA/vector (Jang et al., 1988; Pelletier and Sonenberg, 1988; Thompson, 2012). However, we believe that the polycistronic expression based on the 2A self-processing has some advantages, as compared with the IRES expression system. A minimum nucleotide length of IRES is 10-fold longer than that of the 2A sequence (about 550–600 nt and 60 nt, respectively) and this fact allows to minimize the size of multiple protein expression vector. Another advantage is translation mechanisms for multiple protein expression. IRES is based on the re-entry of ribosomal complex onto an mRNA, whereas the self-processing of 2A/2A-like sequence is thought to progress without dissociation or re-entry of ribosome for downstream translation (Donnelly et al., 2001b). This serial translation of self-processing 2A/2A-like sequence would better equalize the translation rates of upstream and downstream CDSs.

Another advantage of the polycistronic expression system is the saving of cellular transcription- and translation-related factors required for expression of multiple proteins. For example, when RdRp and NP were coexpressed from the equimolar ratio of four monocistronic expression vectors, the level of NP expression was markedly reduced, possibly due to the shortage of cellular RNA polymerase II-related factors (**Figure 2D**, lane 2). The use of a polycistronic vector could halve the kind of protein expression vectors and eliminate the shortage of cellular transcription- and translation-related factors for their expression (**Figure 2D**, lanes 3–8). Therefore, if a large quantity of multi-subunit complexes is wanted as materials for some experiments, it is better to express the subunits polycistronically, but the molar difference of proteins in such a complex should be taken into account, as we expressed the RdRp subunits and NP from different vectors (**Figure 2A**).

The other beneficial use of the self-processing 2A-based expression system is to ensure expression of all the upstream CDSs when a drug-resistant gene is placed at the most downstream in a polycistronic ORF (e.g., **Figure 1B**, 1a2osHygR ORF). The selection of drug-resistant cells allows us to establish stable cells expressing all the genes we want. The longer an ORF becomes by connecting multiple CDSs, the more frequently a disruption of the ORF occurs, especially when the polycistronic expression vector plasmid integrates into the host genome. If a drug-resistant gene is present at the most downstream in the polycistronic ORF, one can select the cells surely expressing all the upstream genes in the presence of the drug. But if such a selective marker is located in another locus and the ORF of interest destroyed or silenced, one cannot eliminate such cells by drug resistance screening. By this approach, we obtained stable cells, almost all of which expressed fluorescent proteins and NP, without cell cloning (**Figure 4A**, HygB, AcGFP- and NP-HygR).

### Drawbacks and Limitations of the Self-processing 2A-Based Polycistronic Expression

The major drawback of this expression system is that the TaV2A-derived sequences remain at the ends of the polycistronically expressed proteins. In some cases, the addition of one proline at the N-terminal end of downstream protein and/or the processed 2A/2A-like peptide at the C-terminal end of upstream protein result in the loss of the protein function. If the N- and C-terminal ends of an expressed protein need to be kept intact, the location of the CDS is limited to the most upstream and the most downstream of polycistronic ORF, respectively. If both ends need to be intact, the protein cannot be expressed polycistronically using a self-processing 2A-based vector. It has also reported that in a certain combination of proteins (e.g., endoplasmic reticulum-targeting protein and protein lacking any signal sequence) the CDS order is limited in a polycistronic ORF (de Felipe and Ryan, 2004).

In this study, when the PB1 CDS was placed at the downstream, the reconstitution of functional RdRp was inefficient (**Figure 2E**), despite similar levels of PB1 expression (**Figure 2D**), suggesting that not only PB1 expression level but also its N-terminal end residues were important for the RdRp activity. The TaV2A-derived proline residue at the N-terminal end of downstream PB1 may have caused a reduction in the RdRp activity. Many studies have suggested that the PB1 N-terminal region is essential for binding to the PA C-terminal region (Gonzalez et al., 1996; Toyoda et al., 1996; Perez and Donis, 2001; Ghanem et al., 2007) and structural studies have reported that the PB1 N-terminal end is inserted into the cavity of PA C-terminal domain (He et al., 2008; Obayashi et al., 2008). In this polycistronic expression system, downstream protein has M1P substitution (**Figure 1A**), suggesting that in our case, the first proline residue at the N-terminal end of PB1 may have a deleterious effect on PB1-PA binding or the overall structure of RdRp even though the first methionine of PB1 originally penetrates the cavity and is exposed to solvent without forming strong interactions with PA residues.

We also encountered the instability of the polycistronically expressed protein. For example, the levels of polycistronically expressed PB2 and PA were very low in many cases (**Figure 2D**), whereas the levels of PB1 was comparable regardless of its CDS location in the ORF. We found that harboring TaV2A-derived sequences made at least PA more sensitive to the proteasomal degradation (**Figure 3B**). Thus, it is necessary to consider the CDS order and the terminal structure when proteins are expressed in this expression system.

By using X-HygR ORF vector, we obtained stable cells expressing the RdRp subunits, and more easily those expressing AcGFP and NP (**Figure 4A**, HygB), suggesting that the number and the kind of proteins expressed may influence to the acquisition efficiency of stably expressing cells. We found that when all RdRp subunits were constitutively expressed, the growth of the host cells was impaired or inhibited. We speculated that transcriptional repression of a polycistronic RdRp-HygR ORF caused the loss of drug resistance. Alternatively, expression of RdRp might be somehow stress and possibly have induced abnormal cell morphology and cell death (**Supplementary Figure S5A**), resulting in the elimination of cells expressing high levels of RdRp.

**FIGURE 6:**
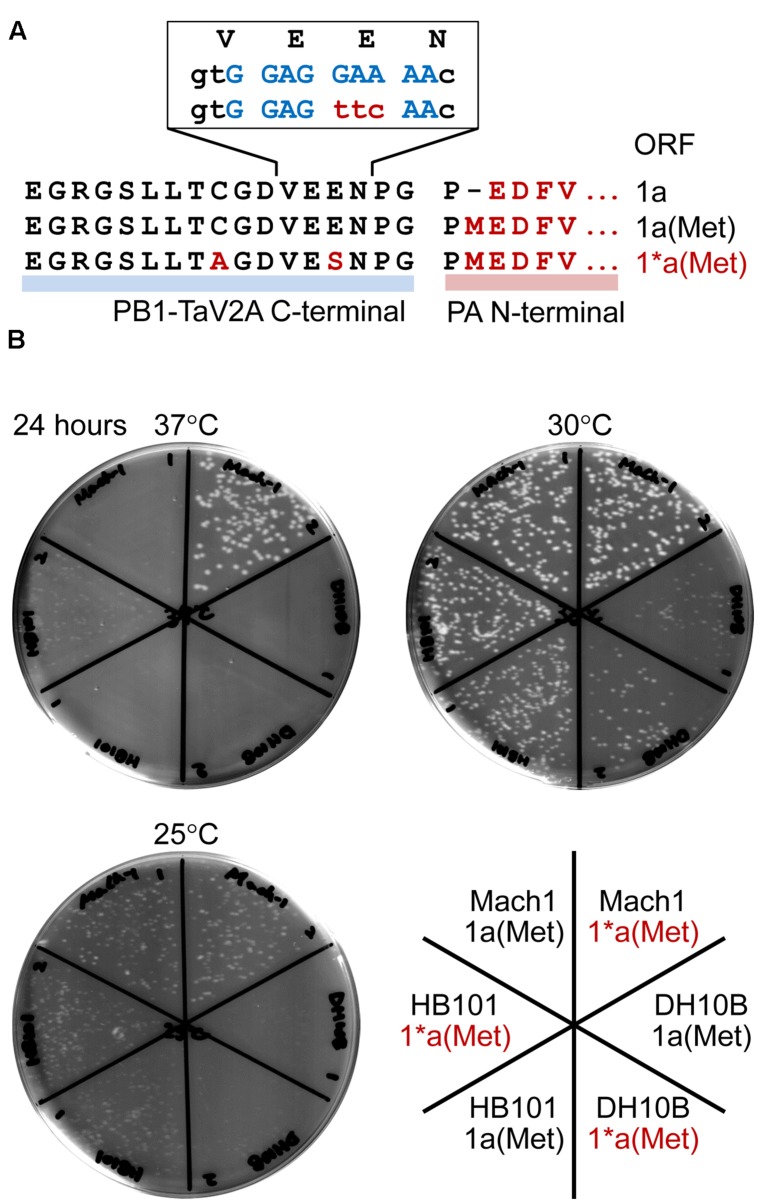
**Temperature-sensitivity of *Escherichia coli* strains transformed with bicistronic PB1-PA expression vectors.** **(A)** Representation of the TaV2A sequences of 1a, 1a(Met), and 1^∗^a(Met) ORF vectors. The N-terminal sequence of PA (downstream region) and modified TaV2A residues [1^∗^a(Met), upstream region] are indicated in red (uppercase letters). A putative SD sequence is indicated in blue, and nucleotide substitutions in the 1^∗^a(Met) ORF are indicated in red (lowercase letters). **(B)** Colony forming assay of transformants at three different temperatures. Mach1, DH10B, and HB101 strains of *E. coli* were transformed with original phCMV-1a(Met) or -1^∗^a(Met) in which the putative SD sequence was disrupted by nucleotide substitution. After recovery in SOC medium, cells were spread on LB agar plates containing kanamycin and incubated at 25, 30, or 37°C for 24 h.

Another technical disadvantage is that a leaky expression of downstream proteins often occurs in *E. coli* cells transformed with TaV2A-based polycistronic expression vector. In our study, the *E. coli* HB101 strain, when transformed with the polycistronic expression vector that contained PA CDS with its start codon at the downstream of TaV2A CDS [e.g., bicistronic 1a(Met) ORF vector], displayed a temperature-sensitive phenotype (**Figure 6B**). Constitutive expression of target proteins in *E. coli* requires a prokaryotic promoter and a ribosome binding sequence such as a Shine–Dalgarno (SD) sequence (Shine and Dalgarno, 1974) immediately upstream of the polycistronic ORF, neither of which is present at a cloning site for target gene in mammalian expression vectors (**Figure 1C**). In this TaV2A-based expression system, however, continuous nine purine bases are present in the TaV2A CDS, are possibly functioned as a SD sequence for a start codon of downstream CDS (Romero and Garcia, 1991; Blattner et al., 1997) (**Figure 6A**).

A number of viruses have self-processing 2A/2A-like sequences (conserved sequence, D*X*_1_E*X*_2_NPGP) (Donnelly et al., 2001a). Like porcine teschovirus and TaV, if the codon of *X*_2_ residue is encoded by GAR (glutamate/E codons, R = A or G), AGR (two of six arginine/R codons), AAR (lysine/K codons), or GGR (two of four glycin/G codons), the region is a putative SD sequence. In contrast, the foot-and-mouth disease virus 2A self-processing sequence can be ruled out (*X*_2_ = S). As shown in this study, the leaky expression of target protein sometimes presents lethal or severe temperature-sensitive phenotypes of host *E. coli*, and the followings can be workarounds: culture at low temperature, use of different host strain, elimination of a putative/original start codon of downstream CDS (e.g., M1P substitution in this study, **Figure 1A**), and/or the inactivation of a putative SD sequence by nucleotide substitutions. We constructed the 1^∗^a(Met) ORF, in which TaV2A peptide had E14S and C9A substitutions, to disrupt a putative SD sequence (**Figure 6A**, non-conserved glutamate codon “GAA” was substituted to a serine codon “TTC”) and improved the antigenicity against our anti-TaV2Apep polyclonal antibody (**Supplementary Figure S2A**, see anti-TaV2Apep epitope). This modification improved the growth of transformed *E. coli*, especially the Mach1 strain (the W strain derivative), at 37°C (**Figure 6B**, 37°C, compare 1a(Met) and 1^∗^a(Met) of *E. coli* Mach1 strain).

## Author Contributions

FM and YM conceived and designed the experiments, and drafted the manuscript. FM performed the experiments and analyzed the data.

## Conflict of Interest Statement

The authors declare that the research was conducted in the absence of any commercial or financial relationships that could be construed as a potential conflict of interest.
