# Supplementary material for: Polycistronic Expression of the Influenza A Virus RNA-Dependent RNA Polymerase by Using the Thosea asigna Virus 2A-Like Self-Processing Sequence
Source: Front Microbiol. 2016 Mar 8;7:288. doi: 10.3389/fmicb.2016.00288 (PMC4782009; doi:10.3389/fmicb.2016.00288)
Supplement: Supplementary file 1 [file Presentation_1.PDF]

Supplementary Material

Polycistronic Expression of the Influenza A Virus RNA-dependent RNA Polymerase by Using the Thosea asigna Virus 2A-like Self-processing Sequence

Fumitaka Momose*, **Yuko Morikawa**

*** Correspondence:** Fumitaka Momose: fmomose@lisci.kitasato-u.ac.jp

This file contains supplementary 5 figures and 2 tables.

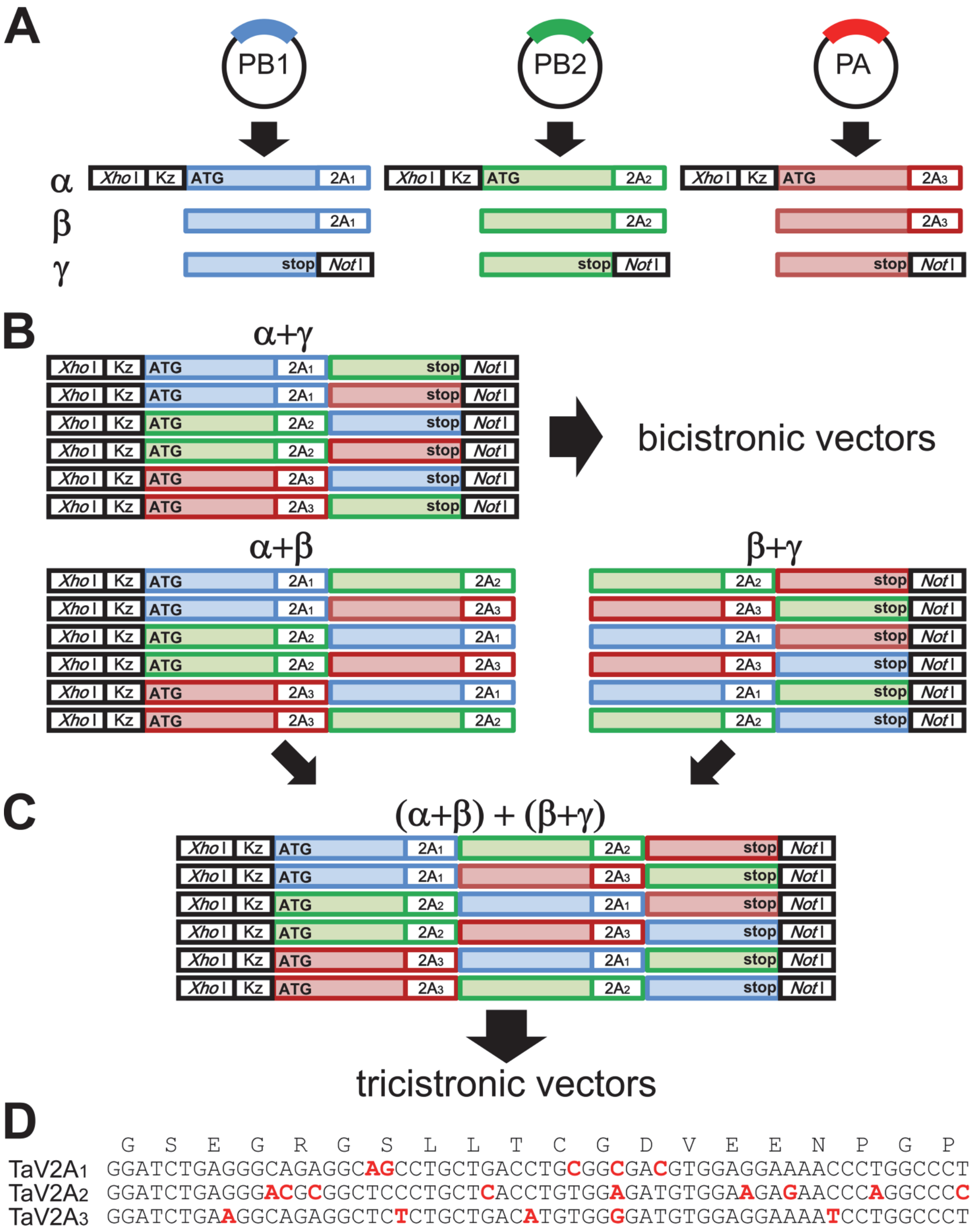

Supplementary Figure 1. Construction scheme of polycistronic expression vectors. See Materials and Methods section for details. **(A)** Amplification of CDS fragments of RdRp subunits. Differences in the upstream and downstream termini are shown. **(B)** Concatenation of two CDS fragments in various combinations. Following ligation of the fragments amplified in (A), concatenation PCR was carried out using the most upstream and downstream primers. **(C)** Concatenation of three CDS fragments in various CDS orders. Overlap PCR was carried out by using α+β and β+γ fragments as templates. **(D)** The CDSs of TaV2A peptide used in this study. These nucleotide sequences are derived from pDON-5 OKSLN (TaKaRa). The differences in each CDS were shown in red.

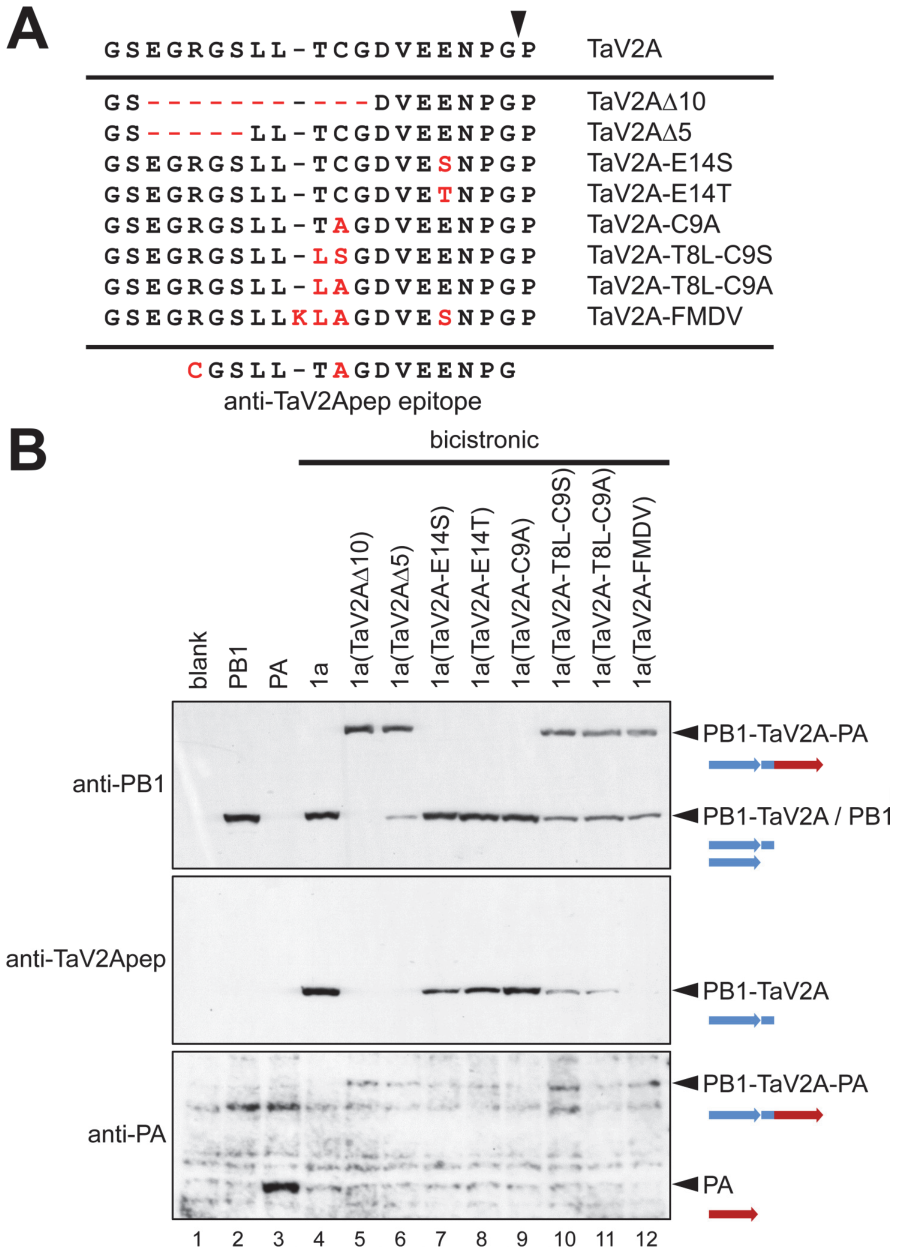

Supplementary Figure 2. Processing of modified TaV2A sequences and expression of PB1 and PA. (A) Representation of TaV2A peptide mutants used in this study. The original TaV2A peptide sequence following a GS linker is shown at the top and the processing site is indicated as an arrowhead. Deletions (- in TaV2AΔ10 and TaV2AΔ5), an insertion (K in TaV2A-FMDV), and substitutions (R3C, T8L, C9A/S, and E14S/T) are indicated in red. The antigen sequence of anti-TaV2Apep is also shown at the bottom. **(B)** Processing and expression of the upstream PB1 and downstream PA. HEK293T cells were transfected with the bicistronic 1a ORF vector (lane 4) and its mutants (lanes 5-12) and were subjected to Western blotting using anti-PB1, -PA antisera, and anti-TaV2Apep antibody. PB1 and PA were also monocistronically expressed (lanes 2 and 3, respectively). Our anti-TaV2Apep antibody does not detect unprocessed form of TaV2A peptide (lanes 10 and 11, PB1-TaV2A-PA).

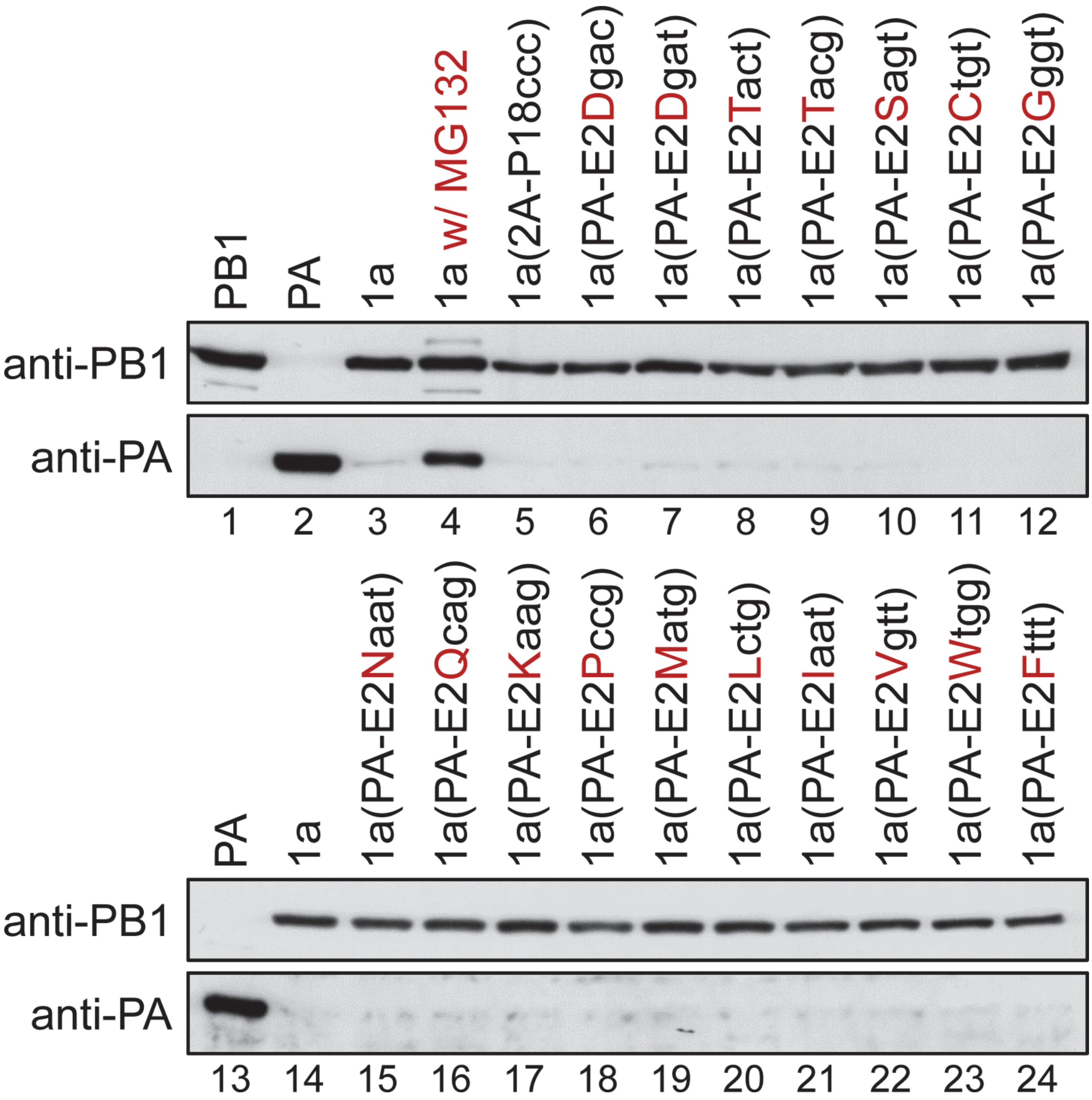

Supplementary Figure 3. Expression of the upstream PB1 and downstream PA from the 1a ORF vector and its derivatives. HEK293T cells were transfected with the bicistronic 1a ORF vector (lanes 3, 4, and 14) and its derivatives (lanes 5-12 and 15-24) and protein expression was analyzed by Western blotting using anti-PB1 and -PA antisera. PB1 (lane 1) and PA (lanes 2 and 13) were monocistronically expressed as positive controls. Cells were transfected with the 1a ORF vector and were cultured in the presence of MG132 (lane 4). The codon sequence of each substitution point is also shown.

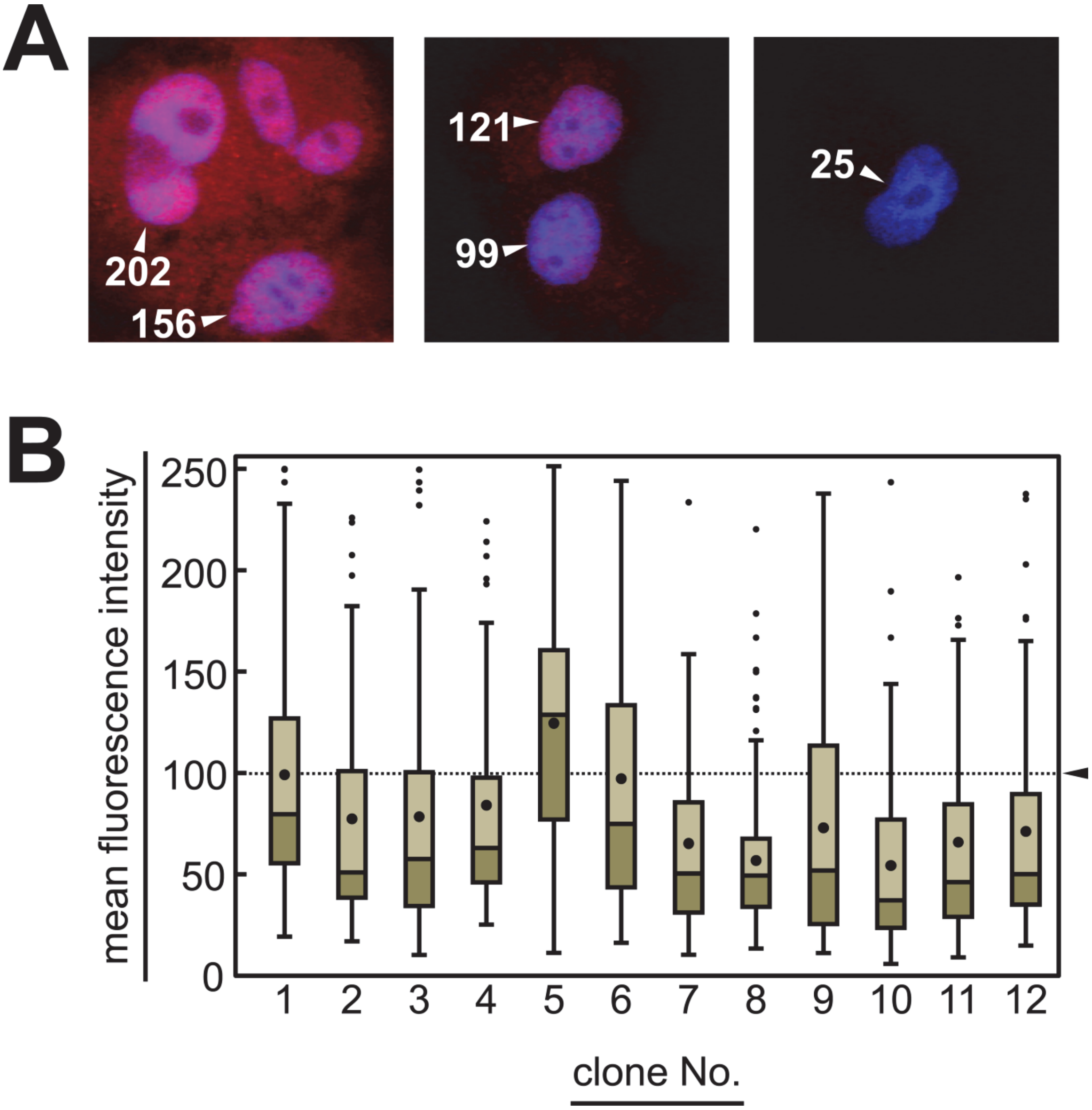

Supplementary Figure 4. The variation of the RdRp expression level in each cell clone harboring the 1a2osHygR ORF. (A) Fluorescence images showing the unequal expression of RdRp. MDCK cell clones harboring the 1a2osHygR ORF were immunostained with anti-TaV2Apep antibody and the fluorescence intensities for the TaV2A peptide (red) within each cell nucleus (blue) were measured. MFI ranging 0 to 255 are shown with arrowheads. A visual detection limit is estimated as MFI = 100. **(B)** The variation of RdRp expression levels in individual cells of each MDCK clone. A box-and-whisker plot represents the distributions of MFI in approximately 50 cells of each clone (No. 1-12). The boxes represent an interquartile range. Averages and medians of MFI are shown as circles and horizontal lines in the boxes, respectively. The whiskers indicate the minimum and maximum MFIs in the range of 1.5 × interquartile range from the box. Outliers are plotted as individual points. An arrowhead and a dotted line show a visual detection limit (MFI =100).

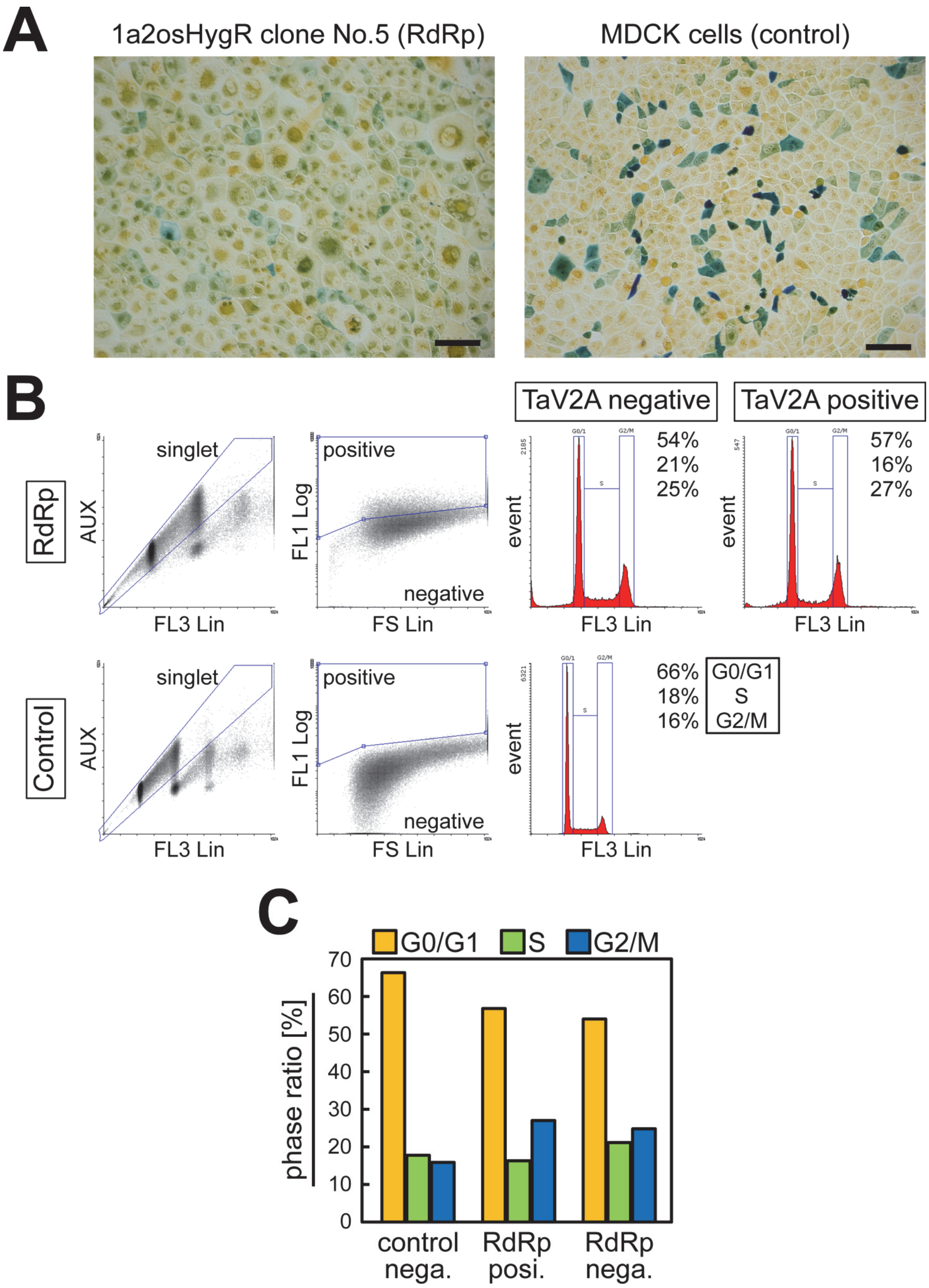

Supplementary Figure 5. Cell-cycle phase distribution and cell morphology of a stable cell line expressing RdRp. (A) Cell-Clock assay. RdRp-expressing MDCK cells (left, 1a2osHygR clone No.5) and original MDCK cells (right) were stained with Cell-Clock dye and were observed with a bright-field microscope. Bars = 100 μm. **(B)** Cell cycle analysis by flow cytometry. The 1a2osHygR clone No.5 (RdRp) and original MDCK cells (control) were stained with PI. Details of the analysis are described in the Materials and Methods. The cell cycle distribution in the G0/G1, S, and G2/M phases (top, middle, and bottom, respectively) is shown with each histogram of the PI intensity (FL3 Lin). **(C)** The phase ratios are also shown as a graph.

Supplementary Table 1. qPCR primer sequences and their hybridization sites.

Target segment	Forward primer hybridization site: Position [a]	Forward primer hybridization site: Sequence [b]	Amplicon length (bp)	Reverce primer hybridization site: Sequence [b]	Reverce primer hybridization site: Position [a]
1	649	E R E L V R K T<br>GAGAGAGAACTGGTCCGCAAAACG<br>GAGAGAGAgCTcGTgCGgAAgACc	140	P G G E V R N D<br>CAGGAGGGGAAGTGAGGAATGATGA<br>CtGGtGGcGAgGTaAGGAATGATGA	788
2	1277	L G V S I L N L<br>TATTAGGCGTCTCCATCCTGAATCTTG<br>TATTAGGCGTgTCgATCCTcAActTgG	145	I Q A G V D R<br>GATTCAAGCCGGAGTCGACAGGTT<br>cATaCAgGCaGGgGTCGACAGGTT	1421
3	640	E I T G T M R K<br>GAAATCACAGGAACAATGCGCAAGC<br>GAAATCACcGGgACgATGaGgAAaC	136	E G K L S Q M S<br>GAGGGCAAGCTGTCTCAAATGTCCA<br>GAaGGgAAaCTcTCgCAgATGTCCA	775
4	873	G I I T S N A<br>GGCATCATCACCTCAAACGCATC<br>GGCATCATtACgTCgAAtGCgTC	152	V R S A K L R<br>CGTCAGGAGTGCCAAATTGAGGAT<br>tGTacGaAGcGCgAAATTGAGGAT	1024
5	403	W R Q A N N G D<br>TGGCGCCAAGCTAATAATGGTGAC<br>TGGCGCCAAGCaAAcAATGGaGAt	150	R M C S L M Q G<br>CAGGATGTGCTCTCTGATGCAAGGT<br>gcGtATGTGtTCgCTGATGCAAGGT	552
6	809	C S C Y P D T G<br>ATGTTCCTGTTACCCTGATACCGGC<br>ATGTTCCTGTTAtCCgGAcACgGGt	141	C S G V F G D<br>GCAGTGGGGTTTTCGGTGACAA<br>GtAGcGGcGTgTTtGGTGACAA	949
7	336	R E I T F H G A<br>AGAGGGAGATAACATTCCATGGGGC<br>AGAGGGAGATcACgTTtCAcGGaGC	148	G L V C A T C E<br>GCCTGGTATGTGCAACCTGTGAACA<br>GatTaGTgTGcGCgACCTGTGAACA	483
8	353	K V A G P L C I<br>GAAAGTGGCAGGCCCTCTTTGTATC<br>GAAAGTGGCAGGaCCctTgTGcATa	150	E E G A I V G<br>GAAGAGGGAGCAATTGTTGGCGA<br>GAgGAaGGtGCgATaGTTGGCGA	502

[a] Nucleotide position of the 5' end of qPCR primer on the target segment cRNA/cDNA.
[b] All nucleotide sequences are shown as positive sense DNA. Peptide, original-, and modified-cDNA sequences are shown from up to down. Synonymously substituted bases are indicated as lower cases with underlines.

Supplementary Table 2. The list of PCR primers used for vector construction.

No.	Name [a]	Oligonucleotide Sequence (5' to 3') [b]	Application / Target
P1	XhoI-Kz-PB1-for	CGCTCGAGCCGCCACCATGGATGTCAATCCGACCTTACTTTTCTTA	
P2	XhoI-Kz-PB2-for	CGCTCGAGCCGCCACCATGGAAAGAATAAAAGAACTAAGAAATCTAAT	The upstream ends of alpha fragments.
P3	XhoI-Kz-PA-for	CGCTCGAGCCGCCACCATGGAAGATTTTGTGCGACAATGCTTCAATCCG	
P4	PB1-wo1Met-for	GATGTCAATCCGACCTTACTTTTCTTA	
P5	PB2-wo1Met-for	GAAAGAATAAAAGAACTAAGAAATCTAAT	The upstream ends of beta and gamma fragments.
P6	PA-wo1Met-for	GAAGATTTTGTGCGACAATGCTTCAATCCG	
P7	PB1-TaV2A1-GRGSct-rev	AGGCTGCCTCTGCCCTCAGATCCTTTTTGCCGTCTGAGCTCTTCAAT	
P8	PB2-TaV2A2-GRGSct-rev	AGGGAGCCGCGTCCCTCAGATCCATTGATGGCCATCCGAATTCTTTT	The downstream ends of alpha and beta fragments.
P9	PA-TaV2A3-GRGSct-rev	AGAGAGCCTCTGCCTTCAGATCCACTCAATGCATGTGTAAGGAAGGAGTT	
P10	TaV2A1-gGRGS-rev	AGGGCCAGGGTTTTCCTCCACGTCGCCGCAGGTCAGCAGGCTGCCTCTGCCC	
P11	TaV2A2-gGRGS-rev	GGGGCCTGGGTTCTCTTCCACATCTCCACAGGTGAGCAGGGAGCCGCGTCCC	A part of TaV2A CDSs, used with P7-P9.
P12	TaV2A3-aGRGS-rev	AGGGCCAGGATTTTCCTCCACATCCCCACATGTCAGCAGAGAGCCTCTGCCT	
P13	PB1-STP-NotI-rev	GTGCGGCCGCCTATTTTTGCCGTCTGAGCTCTTCAAT	
P14	PB2-STP-NotI-rev	GTGCGGCCGCCTAATTGATGGCCATCCGAATTCTTTT	The downstream ends of gamma fragments.
P15	PA-STP-NotI-rev	GTGCGGCCGCCTAACTCAATGCATGTGTAAGGAAGGAGTT	
P16	PB2-ST-STP-NotI-Rev	GTGCGGCCGCCTATTTTTCGAACTGCGGGTGGCTCCAAGCGCTATTGATGGCCATCCGAATTCTTTT	The downstream end of PB2 CDS followed by the Strep tag II CDS.
P17	StrepXchgOne-R1	GCGGAACCTCCCGATCCACCTCCGGAACCTCCACCTTTTTCGAACTGCGGGTGGCT	A part of the Twin-Strep tag (formerly known as the One-STrEP tag) CDS, used with P16.
P18	StrepXchgOne-R2	GTGCGGCCGCCTACTTCTCAAATTGGGGATGAGACCACGCGGAACCTCCCGA	
P19	IFhCMV-XhoKzATG-For	CTTAACAGATCTCGAGCCGCCACCATG	The upstream end of insert DNA fragment, used for phCMV-1a2osHygR construction.
P20	Os-TaV2A2-GRGSct-rev	AGGGAGCCGCGTCCCTCAGATCCCTTCTCAAATTGGGGATGAGACCA	A part of the Twin-Strep tag CDS followed by TaV2A CDS, used for phCMV-1a2osHygR construction.
P21	IFTaV2A2ENPGP-HygR-F	GAGAACCCAGGCCCCATGAAAAAGCCTGAACTCACCG	The upstream end of HygR CDS fragment, used for phCMV-1a2osHygR construction.
P22	IFhCMV-NotStpHygR-Rev	TCTAGAGTCGCGGCCGCCTAGTTAGCCTCCCCCATCTCC	The downstream end of HygR CDS fragment, used for phCMV-1a2osHygR construction.
P23	IFphCMV-KzUp-Rev	CATGGTGGCGGCTCGAGATCTGT	Used for phCMV-HygR vector fragment amplification. phCMV-1a2osHygR was used as a template.
P24	IFTaV2A2-GSEG-For	GGATCTGAGGGACGCGGCTCCCTGCTCA	
P25	IFphCMV-PB2-For	CGAGCCGCCACCATGGAAAGAATAAAAGAACT	
P26	IFTaV2A2GSEG-PB2-Rev	GCGTCCCTCAGATCCATTGATGGCCATCCGAATTC	
P27	IFphCMV-PB1-For	CGAGCCGCCACCATGGATGTCAATCCGACCTT	
P28	IFTaV2A2GSEG-PB1-Rev	GCGTCCCTCAGATCCTTTTTGCCGTCTGAGCTCTT	
P29	IFphCMV-PA-For	CGAGCCGCCACCATGGAAGATTTTGTGCGACA	Used for the amplification of insert fragments to construct phCMV-X-HygR vectors.
P30	IFTaV2A2GSEG-PA-Rev	GCGTCCCTCAGATCCACTCAATGCATGTGTAAGGA	
P31	IFphCMV-NP-For	CGAGCCGCCACCATGGCGTCCCAAGGCACCAA	
P32	IFTaV2A2GSEG-NP-Rev	GCGTCCCTCAGATCCATTGTCGTACTCCTCTGCAT	
P33	IFphCMV-AcGFP-For	CGAGCCGCCACCATGGTGAGCAAGGGCGCCGA	
P34	IFTaV2A2GSEG-AcGFP-Rev	GCGTCCCTCAGATCCCTTGTACAGCTCATCCATGC	
P35	IF-f1OriKpnMut-F	GTCGAGGTACCGTAAAGCACTAAATC	Point substitution primers used for the construction of phCMV1-f1KPN
P36	IF-f1OriKpnMut-R	TTACGGTACCTCGACCCCAAAAAAC	
P37	IF-HumP1P-cRNA5_4G	GGCCGCCGGGTTATTAGCGAAAGCAGG	
P38	IF-HumP1P-cRNA5_4A	GGCCGCCGGGTTATTAGCAAAAGCAGG	Used for the cRNA segment expression vector construction. P37, Seg. 1-3, and 7; P38, Seg. 4-6, and 8; P39, Seg. 1-8.
P39	IF-MusP1T-cRNA3_6T	CGAAGTTGGGGGGGAGTAGAAACAAGG	

[a] Primers containing "IF" in its name were used for the In-Fusion cloning.

[b] If necessary, 5 '-phosphorylated primers were used.
